# UW Supplementation with AP39 Improves Liver Viability Following Static Cold Storage

**DOI:** 10.21203/rs.3.rs-4487319/v1

**Published:** 2024-06-11

**Authors:** S Taggart McLean, Saige Holkup, Alexandra Tchir, Mohammadreza Mojoudi, Madeeha Hassan, Christopher Taveras, S Ozgur Ozge, F Markmann James, Heidi Yeh, Korkut Uygun, Alban Longchamp

**Affiliations:** Massachusetts General Hospital, Harvard Medical School; Massachusetts General Hospital, Harvard Medical School; Massachusetts General Hospital, Harvard Medical School; Massachusetts General Hospital, Harvard Medical School; Massachusetts General Hospital, Harvard Medical School; Massachusetts General Hospital, Harvard Medical School; Massachusetts General Hospital, Harvard Medical School; University of Pennsylvania; Massachusetts General Hospital, Harvard Medical School; Massachusetts General Hospital, Harvard Medical School; Massachusetts General Hospital, Harvard Medical School

## Abstract

Static cold storage of donor livers at 4°C incompletely arrests metabolism, ultimately leading to decreases in ATP levels, oxidative stress, cell death, and organ failure. Hydrogen Sulfide (H_2_S) is an endogenously produced gas, previously demonstrated to reduce oxidative stress, reduce ATP depletion, and protect from ischemia and reperfusion injury. H_2_S is difficult to administer due to its rapid release curve, resulting in cellular death at high concentrations. AP39, a mitochondrially targeted, slow-release H_2_S donor, has been shown to reduce ischemia-reperfusion injury in hearts and kidneys. Thus, we investigated whether the addition of AP39 during 3-day static cold storage can improve liver graft viability. At the end of storage, livers underwent six hours of acellular normothermic machine perfusion, a model of transplantation. During simulated transplantation, livers stored with AP39 showed reduced resistance, reduced cellular damage (ALT and AST), and reduced apoptosis. Additionally, bile production and glucose, as well as energy charge were improved by the addition of AP39. These results indicate that AP39 supplementation improves liver viability during static cold storage.

## INTRODUCTION

Liver transplantation is the only viable treatment option for patients in end-stage liver failure. However, its broad application is limited by the number of available donor organs [1]. A significant limiting factor to the expansion of the donor pool is the loss of viability occurring during transport/preservation. The duration of ischemic cold storage correlates with early allograft dysfunction (EAD) and reduced long-term survival of the grafts (Giwa, S). As a result, thousands of organs are discarded each year (Haugen, C). This clinical problem suggests that better preservation techniques are needed to improve graft quality and help combat the global donor organ shortage crisis [2].

Hydrogen sulfide (H_2_S) is an endogenously produced gaseous molecule through both enzymatic degradation of cysteine via cystathionine γ-lyase (CGL), or non-enzymatic degradation of thiol-containing molecules [3]. H_2_S is proangiogenic, reduces mitochondrial stress, and can regulate the eNOS-NO pathway [4, 5]. H_2_S also has anti-inflammatory and antioxidant properties, and can reversibly inhibit the mitochondrial electron transport chain, thus reducing reactive oxygen species (ROS) formation during reperfusion [6]. During ischemia, H_2_S promotes glucose uptake and glycolytic ATP production [7]. Mice lacking endogenous H_2_S production showed increased damage and mortality following renal ischemia-reperfusion injury, and the introduction of exogenous H_2_S (NaHS) was shown to reverse this effect [8]. Similarly, it has been shown that the introduction of exogenous NaHS in wild-type mice reduces both hepatic and renal ischemia-reperfusion injury [8, 9]. The addition of NaHS to University of Wisconsin solution (UW) preservation solution during SCS reduced necrosis and apoptosis, improving kidney function after transplantation in rats [10]. Despite great success in mitigating the effect of ischemia, NaHS is limited in its application due to the rapid, uncontrollable rate of H_2_S production, resulting in inhibition of mitochondrial electron complexes I and IV and cellular death at high concentrations [11, 12].

AP39 is a mitochondrial-targeting, slow-release H_2_S donor synthesized to improve the mito-protective effects of H_2_S via extended release, and sustained, low-dose release for up to 10 days. Additionally, the introduction of a TPP moiety [13, 14] targets H_2_S at the mitochondria. In a rat kidney transplant model, SCS with 200 nM AP39, resulted in approximately three times increase in survival at 7 days, and increased creatinine clearance [15]. Consistently, the addition of AP39 during porcine kidneys subnormothermic perfusion (21°C) for 4 hours with an O_2_ carrier (Hemopure) improved urine output and graft oxygenation [16]. In renal epithelial cells, the addition of 400 nM AP39 during SCS reduced ROS production [15]. Similarly, in a heterotopic mouse heart transplant model, 200 nM AP39 improved left ventricular ejection fraction and reduced fibrosis following transplantation 24 hours after SCS [17].

**Figure 1.**
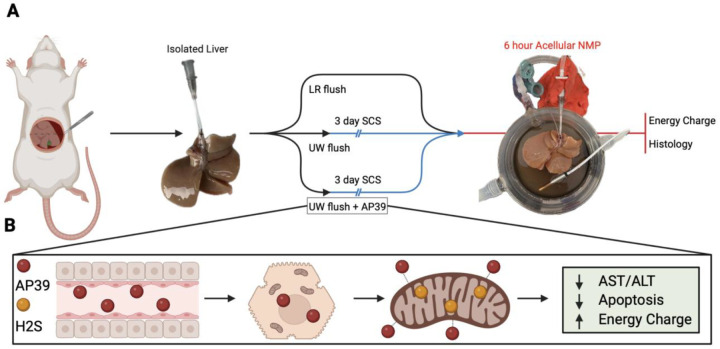
Experimental Outline and Proposed AP39 Mechanism: Experimental schematic detailing the timeline of procurement and perfusion **(A).** Proposed mechanism by which AP39 enters the cell and donates H2S directly to mitochondria **(B).**

In the liver, hepatocytes, make up 20–25% of overall cellular volume. Mitochondria are the main energy source in hepatocytes and are at the center of many of the signaling pathways that mediate hepatocyte injury during ischemia. Thus hypothesized that AP39 supplementation could improve liver viability during SCS. In this study, we tested the benefits of AP39 during liver SCS for 3 days. Following storage, liver viability was evaluated using acellular machine perfusion, allowing real-time assessment of perfusion quality and molecular injury (Fig. 1).

## METHODS

### Liver Procurement

This study is reported in accordance with ARRIVE guidelines. Female Lewis rats (250–300g, Charles River Laboratories, Boston MA, USA) were socially housed in controlled, standard conditions (12-hour light/day cycle, 12C, 30–70% humidity, pathogen-free HEPA filtered ventilated cages, mixed paper/cellulose bedding). All rats had unfettered access to sterile water and chow, as in accordance with National Research Council Guidelines. All rats were cared for by the Massachusetts General Hospital (MGH) Center for Comparative Medicine (CCM). The experimental protocol was approved by the Institutional Care and Use Committee (IACUC) of MGH (Protocol #2011N000111), and all experiments were performed in accordance with established guidelines. Livers were procured as previously described [18]. Briefly, donor rats were anesthetized under 3% isoflurane and maintained at 1%. A transverse abdominal incision was made and the ligaments connecting the superior and inferior portions of the liver were dissected. The gastric and splenic branches of the portal, as well as the hepatic artery, were ligated with 6 − 0 silk (Fine Science Tools inc, Foster City CA, USA). The bile duct was then partially dissected and cannulated with PE-10 tubing (Fisher Scientific). 0.1 U/g heparin was injected into the inferior vena cava through a 30G insulin syringe (Westnet, Canton MA, USA). 5 minutes later, the portal vein was cannulated with a 16G cannula (Westnet), and the liver was immediately flushed with 50 mL UW at approximately 10mL/min, either with or without 200 nM AP39. The remaining connective tissue was then dissected, and the liver was freed from the abdomen. The liver was immediately weighed, and subsequently either perfused for 6 hours at 37°C as described below (fresh control, n = 4), or flushed with UW with 200 nM AP39 (MedChemExpress, Monmouth Junction NJ, USA, n = 6) or vehicle (0.13% v/v dichloromethane) and stored on ice in the same respective solution for 3 days.

### Machine Perfusion

Livers were perfused on a homemade machine perfusion system as previously described [19]. Briefly, a roller pump (Masterflex L/S, Vernon Hills IL, USA) circulated perfusate from a 500 mL basin using 16G in and outflow tubing (Masterflex). Before reaching the liver, the circuit entered a double-jacketed oxygenator (Radnoti, Covina CA, USA), followed by a bubble trap (Radnoti). The system was heated to 37°C by a circulating water bath (PolyScience, Niles Il, USA). Inflow perfusate oxygen concentration was maintained between 500–600 mmHg by a 21% O2, 5% CO2, balance N2 tank (Airgas, Radnor PA, USA). The liver intravascular pressure was zeroed according to system pressure using a portable pressure monitor (Sciatica, London ON, Canada), continuously monitored throughout the perfusion. The liver was hand-flushed with 50 mL lactated ringers (Baxter, Deerfield Il, USA), and attached to the system at a flow of 5mL/min. After a short (1–2 min) adjustment period, the flow was rapidly raised to 30 mL/min, maintaining a pressure below 11 mmHg. Outflow samples were collected from the suprahepatic IVC, every 30 minutes, and inflow samples were taken from a side port immediately before the arterial cannula perfusing the liver. Samples were analyzed using a Siemens Rapidpoint 500 (Siemens, Munich, Germany). Oxygen consumption was calculated according to the following equation: OUR = (inflow O2 - outflow O2) * flow rate / initial weight. Resistance was calculated according to the following equation: R = pressure/flow rate/initial weight. Pressure and flow were recorded every 15 minutes for the first 2 hours, and every 30 minutes thereafter. At the end of perfusion, two biopsies were taken from the peripheral left lateral lobe; one of which was stored in 1% formalin, while the other was immediately snap-frozen in liquid nitrogen.

### Perfusate Composition

The perfusate was composed from a base of 500 mL William’s Medium E (WE) (with sodium bicarbonate, without L-glutamine, with phenol red) (Sigma-Aldrich, St. Louis, MO, USA) into which, the following was added: 1% w/v bovine serum albumin (Sigma-Aldrich), 1% v/v sodium heparin (1,000 U/mL) (MGH Pharmacy), 100 uL insulin (MGH Pharmacy), 200 uL hydrocortisone (MGH Pharmacy), and 0.4% v/v penicillin-streptomycin (Thermo Fisher Scientific, Waltham MA, USA).

### ALT and AST Assay

AST and ALT levels were measured using a commercially available colorimetric activity assay (Cayman Chemicals, Ann Arbor MI, USA) according to the manufacturer’s instructions and as previously published [20]. Outflow perfusate from 1, 3, and 6 hours was incubated with LDH enzyme, and the oxidation of NADH was measured over time according to the absorbance at 340 nm.

### Histological Analysis

Histology samples were moved from formalin to 70% ethanol after 24 hours. Sections were stained with hematoxylin and eosin (H&E) and terminal deoxynucleotidyl dUTP nick end labeling (TUNEL) as previously published [21, 22]. Slides were then imaged at 20X on a Nikon Eclipse E800. On H&E slides, liver sinusoidal endothelial cells (LSEC) sloughing and congestion of the portal vein were analyzed. TUNEL staining was quantified using the Weka trainable segmentation plugin in Fiji [23]. Briefly, nuclei were classified as apoptotic if they were stained brown, or alive if they were stained purple. A probability map of live and dead cells was produced and particle count was applied to find the ratio of dead cells to live cells. All tissue processing was performed at the MGH Histology Molecular Pathology Core Facility (Boston, MA, USA).

### Metabolite analysis

Metabolites were analyzed as previously described [24, 25]. Liver samples were crushed in liquid nitrogen, and metabolites were extracted using an established procedure [26]. All mass spectrometry experiments were performed on a Triple TOF 6600 system (AB Sciex) hooked with a Shimadzu HPLC LC20AD (Shimadzu America) system. Compounds were separated on an analytical Luna NH2 column 2 × 150 mm, 3 um, 100Å equipped with a 2.0 × 4 mm guard column (Phenomenex) using the following conditions: mobile phase A − 100% 5mM ammonium acetate in water, adjusted to pH 9.9 with ammonium hydroxide; mobile phase B − 100% acetonitrile (ACN). Briefly, injection was performed at 20% A, followed immediately by a linear gradient to 100% A over 20 min, hold at 100%A for 4 min, drop to 20% A over 1 min and hold for 5mins at 20%A. The flow rate was set at 0.2ml/min; column temperature was 25°C; injection volume was 2 μl, and autosampler temperature was 4°C, with a total runtime of 30 min including mobile phase equilibration. The mass spectrometer was set to acquire TOF MS spectrum followed by a dedicated product ion spectrum in high sensitivity mode for all nine metabolites of interest. This workflow is also referred to as MRMHR by the vendor. MS spectrum dwell time was 250 msecs and each product ion spectrum was 100msecs. All mass spectrometer experiments were performed in positive electrospray ionization mode. The instrument was set to autocalibrate after acquisition of 5 samples. Briefly, autocalibration was performed by injecting 1 μl of a solution containing 0.5 μmolar AMP, 0.3 μmolar GSSG & 0.2 μmolar FAD. Ion source parameters were as follows: nebulizer gas (gas 1) was 50 psi, heater gas (gas 2) was 55 psi, source temperature 450°C, ionspray voltage was 5500 V, mass range for each experiment 100–900 m/z. Once the mass spec data are recorded, MultiQuant 3.0.2 (AB Sciex) software was used for quantitation by generating chromatographic peak areas. Concentrations of metabolites in unknown samples were determined from standard curves constructed for each metabolite in the MultiQuant software. An eight point standard curve was generated each time prior to running samples using a mixture of known concentrations of the metabolites. All compounds eluted between 13 and 22 min.

### Statistical Analysis

Statistical analysis and graphing were performed using Prism 10 version 10.0.3 (GraphPad Software, San Diego CA, USA). All data was analyzed using ordinary one-way ANOVA followed by Tukey’s multiple comparison test to compare groups and determine significance. Data was reported as means with standard deviation, differences were considered significant when p < 0.05.

## RESULTS

### Addition of AP39 to UW Improves Liver Perfusion Following 3 Days of Static Cold

#### Storage

**Figure 2.**
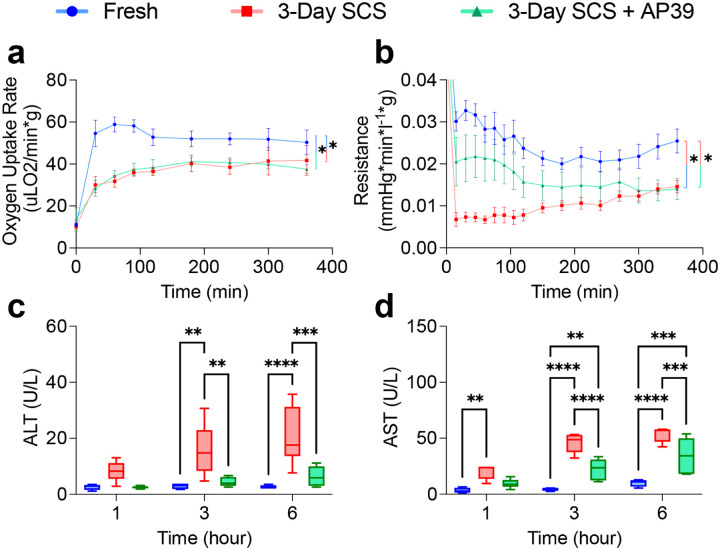
Addition of AP39 to UW Improves Perfusion Parameters Following 3 Days of Static Cold Storage: Comparison of perfusion metrics during acellular normothermic machine perfusion following 3 days of SCS. **(A)** Oxygen consumption was diminished in both SCS groups. (**B**)Resistance in SCS without AP39 was elevated compared to both fresh and SCS + AP39, while no difference was observed between fresh and SCS + AP39. Compared to Fresh and SCS + AP39, **(C)** ALT and **(D)** AST were both elevated. No difference was seen between Fresh and SCS + AP39, although AST was elevated beyond 3 hours. Asterisks denote statistical significance (ordinary one-way ANOVA with multiple comparisons test) *0.01 < *p* < 0.05; **0.001 < *p* < 0.01 ; ***0.0001 < *p* < 0.001 ; ****p < 0.0001. Boxes, median and interquartile range. Whiskers, 5th - 95th percentile. Error bars of line graphs, mean ± S.E.M.

First, we examined the effect of AP39 during SCS. Livers that were immediately harvested (fresh) or stored on ice for 3 days with (AP39) or without AP39 (SCS) were subsequently evaluated during a 6-hour ex-vivo normothermic perfusion, previously successfully employed to model transplantation [27]. After 3 days of SCS oxygen uptake was reduced compared to fresh livers. No difference was observed between livers stored with and without AP39. (Fresh 49.1 uL O2/min*g ± 15.8, SCS 33.9 uL O2/min*g ± 13.1, AP39 34.6 uL O2/min*g ± 11.0, p = 0.9937, Fig. 2a). Importantly, AP39 supplementation reduced vascular resistance (0.019 mmHg*min/L*g ± 0.012, p = 0.0457) compared to SCS (0.027 mmHg*min/L*g ± 0.012), and was comparable to fresh livers (0.012 mmHg*min/L*g ± 0.010, p < 0.0001, Fig. 2b). No difference between the groups was observed in perfusion flow rate or edema at the end of perfusion (Figure S1a-c). Consistently, alanine aminotransferase (ALT, Fig. 2c) and aspartate aminotransferase (AST, Fig. 2d) liver transaminase reflecting cellular injury, were reduced in AP39-treated livers to levels similar to freshly perfused liver. No difference was observed in outflow pH, outflow lactate, or outflow glucose (Figure, S1d-f). All perfusate electrolytes remained within normal range throughout perfusion (Figure S2).

AP39 Improves Hepatocellular Function after Static Cold Storage.

**Figure 3.**
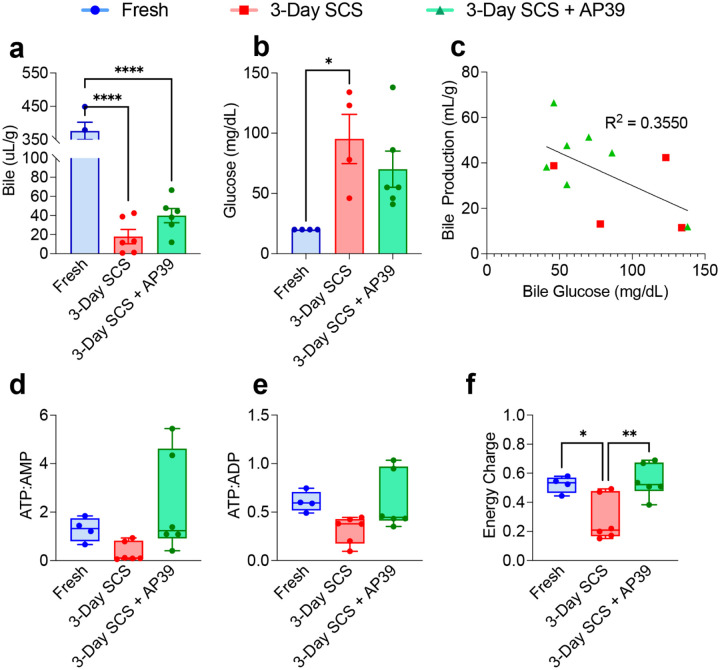
Functional Parameters Show Improved Hepatocellular Functionality when Stored with AP39: Bile was collected through the perfusions and, at the end of experiment, was measured. **(A)** Production was diminished in SCS livers, however, a slight increase was observed in SCS + AP39 compared to SCS. **(B)** When measured for metabolic parameters, bile glucose was elevated SCS compared to Fresh, while no elevation was observed between Fresh and SCS + AP39. (C) When plotting bile production against bile glucose, a weak correlation of r^2^ = 0.355 was observed, although this correlation is limited by the fact that several SCS livers did not produce enough bile for measurement. At the end of perfusion, biopsies were taken and snap frozen in liquid nitrogen, after which key metabolic parameters were measured using LC-MS. The ratio of **(D)** ATP:AMP and **(E)** ATP:ADP was not different between groups, however,

Next we evaluated whether hepatocellular function was improved by AP39 after storage, and during ex-vivo normothermic ex-vivo perfusion. Bile production was similarly reduced in SCS (17.9 uL/g ± 18.3) and AP39 (39.9 uL/g ± 18.2, p = 0.5079) compared to fresh (376.4 uL/g ± 52.2, p < 0.0001 for both) livers (Fig. 3a). However, bile glucose was higher in SCS (95.3 mg/dL ± 40.8) compared to AP39 (70.2 mg/dL ± 36.7, p = 0.4832) and fresh (20 mg/dL ± 0, p = 0.192, Fig. 3b). Interestingly, when plotting bile production, we observed a clear separation between livers stored with or without AP39 (Fig. 3c). We previously demonstrated that graft ATP level correlates with viability [28, 29]. While ATP tended to be higher in the AP39-treated liver, the ratio of ATP:AMP as well as ATP:ADP was similar in all three groups (Fig. 3d, e). However, the energy charge, a calculation based on the ratio of AMP, ADP, and ATP, also used as a marker for graft viability, was higher in AP39 (0.55 ± 0.11) compared to SCS (0.28 ± 0.16, p = 0.0065, Fig. 3f). No difference was observed between SCS and AP39 in other bioenergetic molecules (Figure S3)

### AP39 reduces apoptosis and liver damage

**Figure 4.**
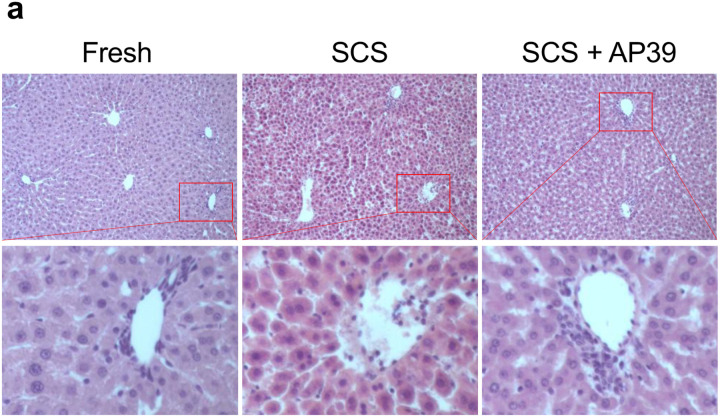
Hematoxylin and Eosin Staining Reveals Improved Liver Microstructure When Stored with AP39: At the end of perfusion, biopsies were taken and formalin fixed, after which they were paraffin embedded and stained with **(A)** hematoxylin and eosin. Microscopic analysis reveals improved cellular morphology in SCS compared to SCS + AP39, with reduced cellular shrinking. Additionally, portal congestion was decreased in SCS + AP39, and endothelial structure was better maintained, showing a similar vascular pattern to Fresh. Asterisks denote statistical significance (ordinary one-way ANOVA with multiple comparisons test) *0.01 < *p* < 0.05; **0.001 < *p* < 0.01 ; ***0.0001 < *p* < 0.001 ; ****p < 0.0001. Boxes, median and interquartile range. Whiskers, 5th - 95th percentile.

**Figure 5.**
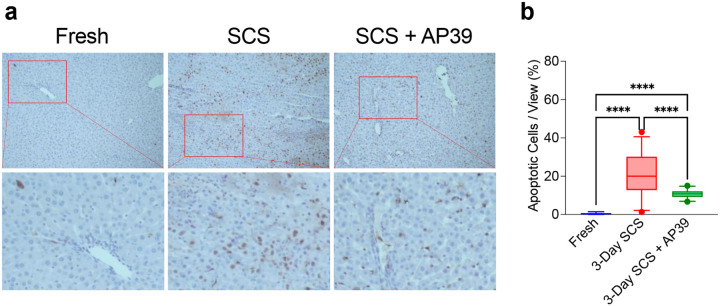
TUNEL Staining Shows Decreased Apoptosis in Livers Stored with AP39: Formalin fixed samples were also stained with terminal deoxynucleotidyl transferase (TUNEL) stain, revealing double-strand DNA breaks, a marker of apoptosis, in brown. **(A)** SCS + AP39 showed a marked reduction in TUNEL positive staining compared to SCS, although still elevated when compared to Fresh. **(B)** Following quantification, SCS without AP39 showed a greatly increased percentage of apoptotic positive cells compared to SCS + AP39. Asterisks denote statistical significance (ordinary one-way ANOVA with multiple comparisons test) *0.01 < *p* < 0.05; **0.001 < *p* < 0.01 ; ***0.0001 < *p* < 0.001 ; ****p < 0.0001. Boxes, median and interquartile range. Whiskers, 5th - 95th percentile.

After simulated transplant, AP39 improved sinusoidal endothelial structure, with reduced sloughing compared to SCS (Fig. 4a). Consistently, hepatocytes showed reduced architectural disruption and hepatocellular shrinkage, suggesting reduced hepatocellular stress (Fig. 4a). Similarly, 3 days of SCS resulted in a 2-fold increase in the number of apoptotic cells compared to AP39 (Fig. 5a SCS 20.7 ± 10.7 vs AP39 10.7 ± 2.1, p < 0.0001 (Fig. 5b) as assessed by TUNEL.

## DISCUSSION

In this study, the addition of the slow-releasing, mitochondrial targeting H_2_S donor AP39 to UW storage solution during SCS reduced post-reperfusion injury and improved cellular function in rat livers. While AP39 was shown to improve heart, kidney, and pancreas function following cold storage, this is the first study investigating its impact during SCS in a liver model [17, 30, 31].

The current clinical limitation for liver storage prior to transplantation is between 9 and 12 hours, restricted by the persistence of metabolism at 4°C, inexorable consumption of cellular energy stores, and ROS production during reperfusion [32]. Surprisingly we observed that oxygen consumption was reduced in livers treated with AP39. Oxygen consumption is a measurement of oxygen extraction during perfusion, shown to correlate with transplant outcomes in rat models [33]. Following cold storage, rat livers are known to exhibit reduced oxygen consumption, which is thought to reflect mitochondrial dysfunction [22, 34]. Similarly, H_2_S transiently inhibits oxygen consumption in the absence of cellular injury and induces a suspended animation state via inhibition of oxidative phosphorylation at complex I and IV [4, 35–37]. Consistently, with the benefit of transition inhibition of oxidative phosphorylation by H_2_S, AP39 was also associated with a reduction in hepatic transaminase (ALT and AST), a surrogate of hepatocellular injury [38]. Additionally, apoptosis was reduced in AP39 treated liver, which might be associated with a reduction in the release of damage-associated molecular patterns following reperfusion [39].

Vascular resistance was improved in livers treated with AP39. Of interest, LSECs are highly susceptible to IR injury [40]. In addition, AP39 was shown to promote vasorelaxation through modulation of NO-signaling; indicating that AP39 may directly improve LSEC outcome following reperfusion [41, 42]. H_2_S has been shown to directly improve LSEC health during sepsis by reducing defenestration [43]. LSEC sloughing is a common indicator of LSEC following reperfusion injury [44]. Similarly, a reduction in LSEC sloughing was observed on H&E. Consistently, portal congestion is commonly observed when blood flow is impaired [45]. Interestingly, a reduction in portal congestion was observed in livers stored with AP39 [46].

Compared to fresh livers, SCS resulted in a reduction in bile production. Although not significant, this was improved by AP39. Of interest, the relationship between bile production and bile glucose demonstrated 2 distinct clusters between livers stored with and without AP39. Potentially, a larger difference and stronger clustering may have been observed if bile glucose were able to be measured for all livers stored without AP39 (no bile production). Consistent with the protective role of AP39, bile production, and quality were associated with both post-transplant liver health and functionality [47, 48]. Additionally, reduction in bile glucose and bile volume during normothermic machine perfusion was previously associated with liver viability [49].

While the direct ratio of ATP:ADP and ATP:AMP was similar in livers stored with AP39, tissue energy charge, previously shown by our team to be associated with transplant outcome [29], was improved by AP39. Consistently, H_2_S is shown to improve post-reperfusion energy stores in rat hearts following warm ischemia [28, 50]. Despite improvements in energy charge, a reduction in NADH:NAD^+^ ratio was observed in livers stored with AP39. NADH has been shown to accumulate during the storage of rat livers as the electron transport chain is compromised, with reduced complex I function [51]. Decreased NAD^+^ levels, when combined with improved energy charge, indicate that following reperfusion, mitochondria with AP39 show improved ATP production [52].

Limitations are acknowledged. First the use of an acellular media strictly limits the IR injury caused by immune response such as neutrophil activation and subsequent parenchymal migration, inducing degranulation and protease activity [53]. Second, despite similarities in microarchitecture between rodent and human livers, several key differences are present such as a reduction in connective tissue in rodent livers, increased perfusate delivery through the portal vein as compared to human livers, and a different surface area to volume ratio [54]. Additionally, the lack of transplantation, and relatively short perfusion period limits our understanding regarding the long-term impact of AP39 supplementation. Future studies should perform further work into elucidating the mechanism by which AP39 ameliorates ischemia-reperfusion-injury in larger human organs and in the context of transplantation.

Altogether this study demonstrates that AP39 supplementation in UW during SCS reduced hepatocellular injury during preservation, improving graft function during simulated transplantation. These results provide the basis for next-generation organ preservation solutions and should be translatable to human livers.

## Data Availability

Data is provided within the manuscript and supplementary information. Additional data are available from the corresponding author by reasonable request.
